# BSR Annual Meeting 2020

**DOI:** 10.5334/jbsr.2327

**Published:** 2020-11-13

**Authors:** Raymond Oyen

**Affiliations:** 1 University Hospitals Leuven, BE

Dear colleagues,

Special circumstances require special measures and initiatives. Covid-19 drastically interfered with all aspects of the society globally and with major repercussion on scientific meetings and continuous education and training.

Given the uncertainty on organization and hosting of future meetings, a few months ago the BSR took the decision to go completely digital with a limited edition of the Annual Meeting 2020.

Thanks to tremendous efforts by the YRS, an attractive scientific program can be offered. Professional support is granted to ensure smooth broadcasting and interaction with participants.

We hope that this exceptional way of organizing the Annual Meeting will nevertheless attract numerous participants.

We now already dare to look forward to the Annual Meeting 2021, hopefully completely “physical” again.

Keep it safe and healthy.

Best regards.

On behalf of the Scientific Committee of the BSR

Raymond Oyen

